# Primary Lymphangioma of the Palatine Tonsil in a 9-Year-Old Boy: A Case Presentation and Literature Review

**DOI:** 10.1155/2016/1505202

**Published:** 2016-10-30

**Authors:** Eleftheria Iliadou, Nektarios Papapetropoulos, Eleftherios Karamatzanis, Panagiotis Saravakos, Konstantinos Saravakos

**Affiliations:** ^1^Department of Otorhinolaryngology, Head and Neck Surgery, Penteli Children Hospital, Athens, Greece; ^2^Department of Otorhinolaryngology, Head and Neck Surgery, Siloah St. Trudpert Hospital, Pforzheim, Germany

## Abstract

Primary lymphangiomas or lymphangiomatous polyps of the palatine tonsil are rare benign lesions that are described infrequently in the literature. The majority of the published cases concern adults. We report a case of a lymphangiomatous lesion of the right palatine tonsil of a 9-year-old boy. Our clinical suspicion was confirmed by the histological examination after tonsillectomy and the diagnosis of primary lymphangioma of the tonsil was made. In this case we discuss the clinical and histopathological features of this lesion and present a short review of the current literature.

## 1. Introduction

Lymphangiomas or lymphagiomatous polyps of the palatine tonsil are rare benign tumors. They present as unilateral or bilateral tonsillar outgrowths and cause a large spectrum of symptoms related to local irritation and airway obstruction [1]. Their pathogenesis has not been clarified and multiple theories have been proposed. Here we report the case of a lymphangioma of the palatine tonsil in a 9-year-old boy and briefly review the existing literature.

## 2. Case Presentation

**Figure 1:**
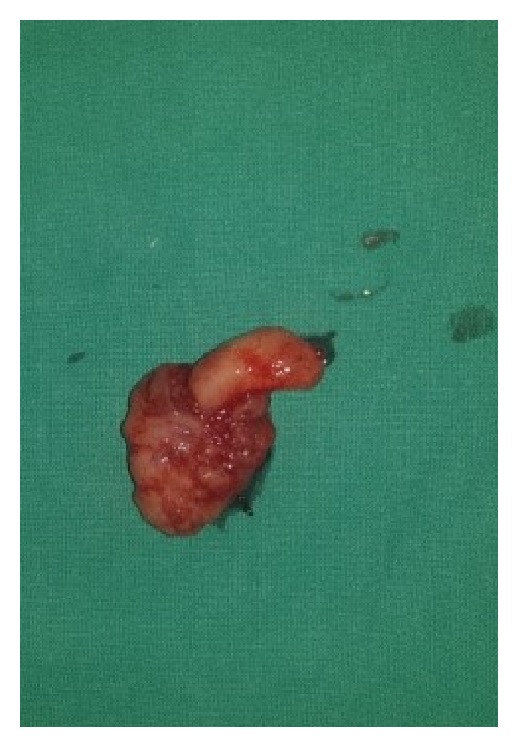
Large oval, pale, pedunculated mass protruding from the lower pole of the right tonsil, compatible to a lymphangioma of the tonsil.

A 9-year-old boy presented to our hospital complaining of a foreign body feeling in the throat of a few months' duration, causing him a nonproductive cough, mild dysphagia, and sleep disturbance. His medical and surgical history were unremarkable. The examination of the oral cavity revealed a large, oval, pale mass protruding from the lower pole of the right tonsil, which was partially obstructing the airway (Figure 1). It was nontender, nonfriable and did not bleed on touch. It was attached to the lower pole of the right tonsil by a narrow elongated stalk. The patient underwent a bilateral tonsillectomy under general anesthesia. The right tonsil and the pedunculated mass were removed by dissection, and hemostasis was performed by ligation. The recovery was uneventful and the patient was discharged the following day. A postoperative follow-up after one month revealed no evidence of any residual or recurrent polypoid disease.﻿

**Figure 2:**
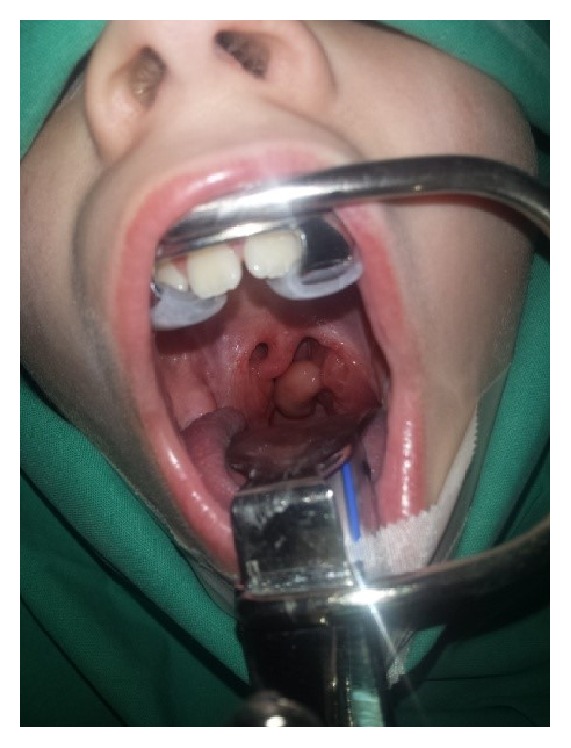
Exophytic polypoid nodule attached to the right tonsil by a stalk (pedunculated polypoid lesion) measuring 1.8 × 1.2 × 1.2 cm.

**Figure 3:**
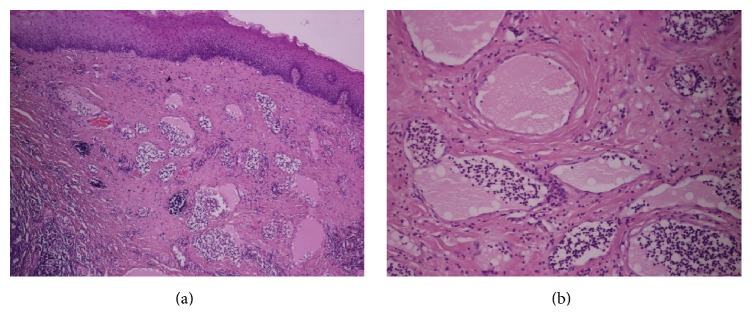
Overlying epithelium without signs of dysplasia. Presence of numerous lymphocytes, with round nuclei and condensed nuclear chromatin, and pedunculated proliferation of vascular channels within abundant fibrous stroma.

The histopathological examination showed macroscopically a tonsil of 3.2 × 2 × 0.8 cm in size with an exophytic polypoid nodule measuring 1.8 × 1.2 × 1.2 cm (Figure 2). Under the microscope, the examination of the mass showed strong cellular proliferation without suspicion of malignancy, consistent with lymphangioma of the tonsil. Abnormal lymphatics did not involve the deep tissues of the tonsil and the tumor was removed in its entirety. The adjacent tonsil showed no significant abnormalities (Figures 3(a) and 3(b)).

## 3. Discussion

More than 90% of all lymphagiomatous lesions occur in the head and neck area, including the cheek, tongue, and floor of mouth [1]. However, lymphagiomatous lesions of the palatine tonsil are rarely reported in the literature [2–18]. The references are even less frequent in the pediatric population [1–3, 5, 9]. Many authors believe that the true incidence may be higher than reported [1, 2, 9].

Histologically, lymphangiomas are formed by abundant dilated lymphatic and blood channels mixed with a fibrous stroma of lymphoid and adipose elements [14]. There are several theories regarding the pathogenesis of this type of tonsillar disorder. The first one suggests that lymphangiomas arise due to sequestration of lymphatic tissue derived from primitive sacs, which retain their rapid and proliferative growth potential but fail to join the major lymph sac of the body. The second theory proposes that it arises from endothelial fibrillar membranes, which sprout from the walls of the cyst, penetrate the surrounding tissue, canalize, and then produce more cysts along lines of least resistance. These cysts maintain their ability to branch out and grow, and they do so in an uncontrolled, disorderly manner with a tendency to penetrate and destroy normal anatomic structures. This uncontrolled proliferation is thought to be caused by a dysregulation of growth factors involved in lymphangiogenesis known as Prox-1 and vascular endothelial factor (VEGF-C) [1]. The third theory advances the hypothesis that the primitive lymphatic sac does not reach the venous system [1]. Finally, there is another pathogenetic theory, which suggests that chronic inflammation of the tonsil and the associated obstruction of the lymphatic channels cause mucosal congestion and subsequently polypoidal swelling [5]. However, this last theory is considerably unlikely because chronic tonsillitis is much more common than the lymphangiomatous tonsillar polyps, and because there are many patients, like our case, who do not have a history of recurrent tonsillitis [12, 16].

The clinical behavior of the tumor is largely unknown, because most of these lesions are diagnosed histologically after surgical excision of the tonsils. Common presenting symptoms include dysphagia, dyspnea, foreign body sensations, sore throat, and chronic tonsillitis. When the mass is very large, it can affect surrounding vital structures to produce rhinolalia clausa, respiratory difficulty, stridor, excessive saliva, or nausea [1]. In all reported cases, the disorder behaved in a benign fashion and no complications or recurrences have been reported. In our case, the main symptoms were foreign body sensation, interrupted sleep, and mild dysphagia, which were resolved after surgery.

The history and the clinical examination are important for the diagnosis, but a histological examination is required to establish the diagnosis. The differential diagnosis includes lymphangiectasia, hemangioma, arteriovenous malformation, fibroepithelial polyps, and papilloma. In our young patient's case, the preoperative suspicion was strong because of the typical appearance and the large size of the lesion. However, as a lymphangioma clinically may resemble a true neoplasm of the palatine tonsil, the lesion needed to be removed in order to complete an accurate histological diagnosis and to rule out malignancy.

Tonsillectomy is the curative procedure of choice for the management of these tonsillar polypoid lesions. An excision of the polypoid mass may be the only necessary procedure, while sclerosing therapy with OK-432 or radiotherapy is not suggested. Furthermore, a carbon dioxide laser has been frequently used to treat a group of pediatric patients with benign lesions of the upper aerodigestive tract exclusive of the larynx, but there have been no benefits compared to the cold steel tonsillectomy [1]. As our patient presented a bilateral tonsillar hypertrophy, we decided to proceed with a traditional bilateral tonsillectomy, with good results.

## 4. Conclusion

In summary, this case demonstrates a case of tonsillar lymphangiomatous polyp occurring in a 9-year-old boy. It is believed that the incidence of the tonsillar lymphangiomatous polyps is greater than what is reported in the literature, especially in the pediatric population. The aim of this case report is to consider this type of benign tumor in the differential diagnosis of tonsillar masses in childhood.
